# Production of Verb Tense in Agrammatic Aphasia: A Meta-Analysis and Further Data

**DOI:** 10.1155/2015/983870

**Published:** 2015-09-20

**Authors:** Yasmeen Faroqi-Shah, Laura Friedman

**Affiliations:** ^1^University of Maryland, College Park, MD, USA; ^2^University of Wisconsin-Madison, Madison, WI, USA

## Abstract

In a majority of languages, the time of an event is expressed by marking tense on the verb. There is substantial evidence that the production of verb tense in sentences is more severely impaired than other functional categories in persons with agrammatic aphasia. The underlying source of this verb tense impairment is less clear, particularly in terms of the relative contribution of conceptual-semantic and processing demands. This study aimed to provide a more precise characterization of verb tense impairment by examining if there is dissociation *within* tenses (due to conceptual-semantic differences) and an effect of experimental task (mediated by processing limitations). Two sources of data were used: a meta-analysis of published research (which yielded 143 datasets) and new data from 16 persons with agrammatic aphasia. Tensed verbs were significantly more impaired than neutral (nonfinite) verbs, but there were no consistent differences between past, present, and future tenses. Overall, tense accuracy was mediated by task, such that picture description task was the most challenging, relative to sentence completion, sentence production priming, and grammaticality judgment. An interaction between task and tense revealed a past tense disadvantage for a sentence production priming task. These findings indicate that verb tense impairment is exacerbated by processing demands of the elicitation task and the conceptual-semantic differences between tenses are too subtle to show differential performance in agrammatism.

## 1. Introduction

Agrammatic aphasia is a cluster of language symptoms following damage to left hemisphere peri-Sylvian regions. The core feature of agrammatic aphasia is severely impoverished sentence production: utterances consist of words strung together in an ungrammatical sequence, or, at best, simple canonical sentences (e.g., subject-verb-object, in English) [1–3]. Associated features of agrammatic aphasia include difficulty with verbs (both in sentences and single word recall) and with grammatical morphemes (both free standing and inflectional morphemes) [4–6]. Further, many (but not all) individuals with agrammatic speech production present with asyntactic comprehension [7]. This refers to difficulty interpreting syntactically complex sentences (such as the passive and object relatives), particularly in semantically reversible contexts. Whereas sentence production impairment is the hallmark of agrammatism, there is considerable individual variability in the extent of other deficits [8, 9].

Crosslinguistically, sentence production difficulty in agrammatism is often characterized by exceptional difficulty producing certain types of morphosyntactic structures, such as tense marking, relative to other structures, such as agreement and mood marking (e.g., in English [10–13]; in Hebrew [14]; in German [15]; but see conflicting results in [16]; in Spanish [17, 18]; in Dutch [19, 20]; and in Greek [21–23]). The crosslinguistic data also demonstrate that the tense disadvantage occurs even when morphological complexity of the verb, such as affixation and additional free grammatical morphemes, is held constant [12, 24]. 

*Accounts of Tense Production Deficits in Agrammatism*. Given the prominence of tense deficits in agrammatism, there are numerous tense-centric theoretical accounts of agrammatic aphasia [12, 14, 15, 19]. Some accounts are based on the syntactic theory of generative grammar [25, 26], according to which certain syntactic nodes hosting complementizers, along with other functional categories, such as tense, are located higher in the syntactic tree than others (e.g., agreement, mood, and aspect). Syntactic accounts, such as the Tree Pruning Hypothesis [14], propose that this hierarchical relation between different morphosyntactic categories is observed in agrammatism such that impairment of any node implies impairment of higher nodes as well (also [27]). However, most crosslinguistic evidence has found that breakdown of functional categories does not consistently follow the pattern of morphosyntactic hierarchy (e.g., [11, 15, 16, 21, 22, 28]).

Another family of tense-centric accounts of agrammatism draws attention to the fact that, in addition to its morphosyntactic role, verb tense interfaces with semantics of the event. That is, tense is an “interpretable” syntactic feature [15]. The tense underspecification account suggests that the tense features (+/− PAST) are inadequately specified in functional category representations of persons with agrammatic aphasia [15]. The diacritical encoding hypothesis uses the framework of language production models and claims difficulties in encoding semantic components of the message onto inflectional morphology [12]. Some of these accounts include difficulties with verb aspect, which also represents the temporal state of an event [21, 22, 29–31]. Support for differentiating between the syntactic and semantic components of tense morphology in agrammatic individuals comes from better performance on knowledge of syntactic well-formedness constraints (e.g.,* is walking* versus ^∗^ *will walking*) than on knowledge of the correspondence between verb forms and their time reference [12, 32]. The argument is that, unlike subject-verb agreement which serves a purely morphosyntactic function, verb tense serves a deictic function because it refers to the temporal relationship between the event being described and the time of speaking [12, 19, 21, 23, 31, 33, 34]. These deictic implications of verb tense confer additional conceptual-semantic complexity in its formulation and comprehension.

Further, it has been proposed that reference to past events is “selectively impaired in agrammatic aphasia” such that past tense and perfect aspect are particularly challenging to produce compared to present tense and imperfect aspect [19, 35]. In the past discourse linking hypothesis (PADILIH), Bastiaanse and colleagues have used the greater temporal mismatch between speaking and event time to explain why their studies found worse performance on sentences eliciting the past compared to the present [19, 29, 36, 37]. Other authors have noted that discourse linking is not restricted to past events and that reference to the future is also discourse linked because it is a projection to a subsequent time point [38–40]. It is currently unclear whether production of past events is actually selectively impaired or worse than present and future events because numerous other studies have reported no accuracy differences between past and present/future [11, 12, 15, 16, 20, 21, 31, 41, 42].

There are other theoretical accounts of agrammatic production that identify more general sources of difficulty. Resource limitation accounts propose an interaction between computational demands and performance success, especially for syntactic computations. Empirical support for the impact of limited processing resources on agrammatic production comes from the influence of task complexity [20] and syntactic complexity ([33]; see [43] for agrammatic comprehension). Kok and colleagues [20] demonstrated the effect of task complexity on successful production of verb morphology by comparing success on two tasks: sentence completion, in which participants had to inflect a nonfinite verb, and anagram ordering, in which individual words had to be sequenced to form a sentence* and* the verb had to be inflected. Agrammatic participants performed significantly worse on the more demanding anagram ordering task, suggesting an effect of computational load in computation of verb morphology.

To summarize, a variety of accounts have been proposed to characterize the difficulty with production of tense morphology in agrammatism. A majority of the more recent accounts incorporate some reference to the semantics of time, one account proposes further dissociation within temporal morphology, and some accounts allow for performance variation based on task processing demands. Although there is substantial empirical data on agrammatic tense production, it is unclear whether tense morphology is modulated by semantic (or any other) variables. Actually, a precise characterization of the verb tense deficit in agrammatism is lacking, which is a precursor to developing a reasonable explanation for this symptom. This study aims to further our understanding of verb tense performance by investigating the influence of two variables: temporal category (past, present, or future) and the elicitation task. A brief background on these two variables is provided before describing the current study. 

*Linguistic and Cognitive Representation of Time*. Most of the world's languages have some mechanism of conveying the unfolding of events over time.* Tense* refers to grammatically expressing the linear temporal relationship between the moment of speaking (S), the moment of the event (E) being described, and, sometimes, another reference point (R) [44–47]. For example, a sentence such as “*He signed the papers*” refers to an event that occurred before the time of speaking as shown in Table 1 (adapted from [45]). This contrasts with simple present, where the speaking and event time are simultaneous, and simple future, where the speaking time occurs prior to the event (see Table 1). Time is also depicted by* aspect*, which refers to the temporal distribution of an event, irrespective of event time [48]. Hence, an event may be completed or perfective as in “*He had signed the papers*/*He has signed the papers*,” or ongoing and imperfective as in “*He was signing the papers*/*He will be signing the papers*” (Table 1). There is considerable variability across and within languages in how tense and aspect are denoted [49]. For example, tense and aspect may be conflated, occurring on the same grammatical morpheme, which is frequently a verb affix; for example, the preterite –*Ó* in Spanish refers to past and perfect. Within a language, some tense/aspectual morphemes may be conjugated on the verb, while others may be free morphemes (often preverbal auxiliaries), such as the contrast between present/past (*verb* +* s*/*verb* +* d*) and future in English (*will* +* verb*) as shown in Table 1. Temporal relationships may also be represented by adverbs, such as “*earlier*” or “*yesterday*.” And in some languages such as Mandarin, adverbs are the only mechanism of denoting temporal relations.

Empirical investigations of tense and aspect processing in neurologically healthy adults have mostly examined the extent to which different sentences evoke a mental representation of the event. For example, Magliano and Schleich [50] presented participants with short stories in which a critical sentence in the middle of the story described an action in past progressive or simple past (*Stephanie was changing/changed the flat tire*). This was followed by a yes/no probe question about the event (*Is Stephanie back on her way to the airport yet?*). Participants' response times to the probe questions were faster for imperfective sentences. Similar findings of faster response times for imperfectives compared to perfectives have been found in sentence-picture matching [51], self-paced reading [52], and action execution [53]. As for tense processing, monitoring for a word following a short paragraph was faster when the target word occurred in sentences with present tense compared to past tense in Spanish [54]. The interaction between tense and aspect was examined using six sentence types (past and present tense in simple, progressive, and perfect aspects) in a sensibility judgment task [55]. Response times were influenced by verb aspect, with the fastest responses to simple aspect (*…closed/closes the drawer*) and slowest responses to perfectives (…had closed/has closed the drawer), but there was no effect of verb tense on response times.

To summarize, data from neurologically healthy adults shows that sentences in the imperfective aspect are consistently faster than perfectives, while present tense is faster than past only when aspect is held constant as in Carreiras et al. [54]. This implies that mental representation of events is constructed more rapidly if the event is construed as ongoing (imperfective) and occasionally if there is temporal overlap between speaking time and event time (present tense). This finding is often explained using the embodied cognition framework, according to which sentence processing evokes sensorimotor simulations, which are probably more vivid for ongoing events [50, 51]. It is also possible that evoking mental representations of completed events (i.e., past/perfective sentences) places additional demands on memory for perceptual details [40]. Using a similar logic of perceptual detail, evoking mental representations of the future (tense) is argued to be more challenging (greater abstraction) because the event has not yet occurred [40, 56, 57]. For example, Pinker's [57] analysis of the semantics of tense differentiates between events that have actually taken place or are ongoing (*realis*) and events that are hypothetical and future (*irrealis*). Empirical findings have been consistent with less robust (or more abstract) mental simulation of future events compared to past and present events: Zwaan et al. [58] found faster self-paced reading times for present and past events compared to future events. A functional neuroimaging study that compared past, present, and future tense sentences in Hebrew found activation of sensorimotor regions for past and present but not for future [40]. Sentences in the future activated ventromedial prefrontal regions, and this contrast between activation instances for present/past versus future sentences mirrored that for concrete versus abstract sentences.

On the basis of these processing differences observed in healthy adults, one could predict that verb tense/aspect with a greater mismatch between speaking, event, and reference times will be more vulnerable to the effects of aphasia (Table 1). In other words, past and future tenses and perfect aspect would be more impaired than present tense and imperfective aspect. Few studies of agrammatism have compared perfect and imperfect aspect to glean the influence of grammatical aspect on sentence production (but see [29]). In some studies, tense and aspect are conflated, where simple past was compared with present progressive, rather than with simple present ([36] in English). In some languages, controlling the aspect made for unnatural sentences for one tense more than other tenses: in Dutch, past tense is typically used with perfect aspect, and requiring participants to use imperfect aspect with past tense was unnatural [20]. In order to examine if conceptual differences (i.e., in the mental representation of temporal reference) can account for tense impairment, it is important to first determine if there is a consistent performance difference between tenses. 

*Influence of Elicitation Task on Performance*. A variety of experimental paradigms have been used to examine performance on tense and other functional categories. The primary motivation behind the various paradigms is to manipulate which specific tense needs to be produced and to indicate this unambiguously to the participant. The various paradigms are also used to circumvent cooccurring limitations in lexical retrieval, word ordering, and working memory. For instance, participants may be required to complete a sentence fragment, the word to be inflected is provided, or multiple words are given in a forced-choice structure [10–12, 15, 59]. This sentence completion paradigm places minimal lexical retrieval demands, hence tapping the morphosyntactic abilities of the person (e.g.,* Yesterday the man…the apple* [eats, will eat, ate]). However, this task is unnatural and does not approximate* sentence production* in its true sense [20]. In the sentence production priming paradigm used in the Test for Assessing Reference of Time (TART, [60]), the tester models the target sentence for a photograph and the participant is required to produce the same sentence structure for a slightly different photograph (*For this picture you can say the man ate the apple. For this picture you can say the man…* [peeled the apple]). In this paradigm, lexical retrieval demands are minimal, while there may be additional demands on working memory compared to the sentence completion task. Notably, the photographs used to elicit past tense in TART often do not portray the target action because the action has already been completed. For example, the photograph of a man looking at an empty plate is used to elicit “*The man ate an apple*.” In contrast, the photographs used to elicit present tense portray the action, which could potentially create confoundedness of greater difficulty for past tense sentences.

A third experimental task provides the content words in random order and requires the participant to produce the sentence; a picture may or may not be used to aid the sentence [20, 42]. This task places additional demands on syntactic formulation of the entire sentence but more closely approaches sentence production. Finally, grammaticality or goodness judgment tasks are used, which tap linguistic competence rather than sentence production [15, 32, 61]. A crucial calculation in the interpretation of grammaticality judgment performance is an estimate of response bias, which refers to the possibility that a participant may push the* yes* or* no* button for most of the trials showing little sensitivity to the construct being tested [62]. Unfortunately, most studies on tense deficits using grammaticality judgment task fail to report measures of response bias (A-prime or D-prime). Further, some studies have only reported findings on the ungrammatical sentences (e.g., [63], for Arabic) while others compute accuracy over both grammatical and ungrammatical sentences. The role of task differences on performance was demonstrated by Kok et al. [20] for a group of Dutch speaking aphasic participants: performance on the same set of sentences was worse when the entire sentence had to be produced compared to sentence completion, in which only the inflected verb had to be produced. Therefore, in order to determine whether true differences exist across tenses, it is important to evaluate whether tense differences exist irrespective of the experimental manipulation.

To summarize, a theoretical account of tense impairment in agrammatism has been elusive. An impairment in the cognitive representation of time or in conveying temporal reference on verb morphology is currently a promising explanation. In order to evaluate this explanation and further our understanding of tense deficits, we need to unambiguously determine whether there are differences in performance across tenses that supersede crosslinguistic and methodological differences. 

*The Present Study*. This study aims to provide a more precise characterization of verb tense impairment in agrammatism by examining whether there is dissociation within tenses such that any one tense is more impaired than other tenses. The basis for this question comes from (1) sentence comprehension findings in healthy adults, showing that a mismatch between the time of speaking and the occurrence of an event (as in past tense and perfect aspect) incurs a processing cost compared to when the speaker refers to an ongoing event (as in present tense and imperfect aspect), (2) recent mixed findings across studies about the relative impairment of past tense compared to other tenses, and (3) the potential for methodological differences to produce different patterns of results across studies. In the present study, we compared whether past, present, and future tenses show differential levels of impairment using two approaches: (1) a meta-analysis of prior studies and (2) reporting new data from a group of 16 persons with agrammatic aphasia using an experimental task that has been relatively less frequently used in prior research. Given that there have been numerous studies of verb tense in agrammatism, it is worth reexamining the substantial corpus of existing data to synthesize the existing findings on tense deficits. It should be noted that although different tenses have been elicited in prior studies, a comparison of tenses was not the primary focus of most prior studies (with the exception of Bastiaanse et al. series using TART). Rather, the focus was on comparing tense with other functional categories (e.g., [10, 14, 16, 28]). Hence, the meta-analysis of prior studies presents these data in a different perspective.

This study posed two research questions. First, we asked whether there is a difference in performance across different tenses (past versus present versus future) and relative to tense-neutral stimuli. Although we initially intended to compare perfect versus imperfect aspect, this question could be not addressed because of the small number of studies reporting aspectual comparisons. Second, we asked whether there is an interaction between elicitation task and tense performance. Based on sentence comprehension data from healthy adults and the differences in speaking and event time for past tense, we hypothesized that past tense performance would be worse than present tense. Additionally, we hypothesized that this difference would be evident in select experimental tasks, such as sentence production, but not in grammaticality judgment, due to the more complex computation demands of production tasks.

## 2. Meta-Analysis of Published Studies

### 2.1. Methods

Published articles in English peer-reviewed journals reporting investigations of functional categories in persons with aphasia were identified using the key words aphasia, morphology, functional categories, tense, aspect, and agrammatism. The electronic databases used for the search were Science Citation Index, Medline (PubMed), PsycInfo, and Academic Search Premier. In addition, citation lists of identified articles were combed for further sources. The search was restricted to research studies available electronically between 1980 and December 2013. This identified approximately 60 potential articles for the review. We read the abstracts of these articles for relevance and excluded several studies based on content, narrowing the number of potential articles down to 38. After reading the text of the remaining articles, we used the following predetermined inclusionary criteria to identify the studies that qualified for the meta-analysis: (1) the study reported original data from participants with a diagnosis of sudden onset aphasia (not progressive); (2) language profile was described in adequate detail to determine the specific symptomatology of the patient (e.g., agrammatic, nonfluent, and fluent); (3) native language performance was reported, although the participants could have been multilingual; (4) the study provided data for individual participants, with breakdown of scores for the various tense types; (5) task/stimuli were presented as sentences (i.e., not single word repetition); (6) syntactically simple sentences were used to minimize the confoundedness of syntactic complexity with tense encoding; (7) the study presented data for some combination of past, present, and future tense stimuli to enable within-subjects comparison (i.e., not just a single tense). Other functional categories such as agreement, mood, and aspect were noted. Reports that duplicated data, such as conference proceedings and full articles, were included only once. Multiple datasets from individual patients were included in the meta-analysis only if each dataset was original to the study. This resulted in a final set of twelve articles, with 106 individual participants totaling to 143 datasets.

#### 2.1.1. Coding and Data Analyses

All studies were coded for language of testing, description of aphasia profile, description of lesion information, the experimental task, response type (e.g., verbal), the number of stimuli used, raw scores, proportion accuracy, and the conclusions of the authors. Four different experimental tasks were used in the studies reviewed: (1) sentence production priming (SPP), in which participants were provided with a pair of pictures (the examiner modeled the target sentence for the first picture and asked the participant to describe the second picture using a sentence similar to the model); (2) sentence completion (SC), in which participants were required to complete a sentence fragment using the correct form of a given word from among a forced choice (e.g.,* Speaks*,* Speak*, and* Spoke*); (3) sentence production using picture description (SPPic), in which participants had to describe a single picture using temporal adverbs as prompts (e.g.,* Yesterday*,* Nowadays*); (4) grammaticality judgment (GJ), in which the participant decided whether a sentence was grammatical or not. The stimuli used in studies were coded for tense into the following four categories: present, past, future, and neutral. Tense-neutral stimuli were verbs that did not require tense marking, because these occurred in embedded clauses or other syntactic structures where tense was marked on a different main verb (e.g., Sheila wanted to* move* to the city). When different aspects were used, such as simple present and present progressive, these were combined into the corresponding tense. For studies where raw scores were not reported, these were computed from the relevant figures or percentage scores. Raw scores were converted to standard (*z*) scores and statistically compared using analysis of variance with tense and task as the independent factors.

### 2.2. Results

The meta-analysis included 143 total datasets elicited from seven different languages. Sixty-eight of these datasets included three tenses (past, present, and future) while 75 datasets included two of the three tenses. Sentence completion task was used for 60 of the datasets, sentence production priming was used for 49 datasets, sentence production with pictures was used for eight datasets, and 26 datasets used grammaticality judgment. The average age of participants was 55.53 years and the average time following onset was 74.85 months. Of the 106 participants, 77 were male, 26 were female, and three participants' gender was not reported. The majority of the patients had a left cerebrovascular accident. Individual participant data from each study are presented in Table 2. Across all tasks, accuracy was 47.7% (SD = 13.2).

Analysis of variance revealed statistically significant main effects of sentence type (*F*(3,392) = 16.78, *p* < 0.001) and task (*F*(3,382) = 9.35, *p* < 0.001). There was no significant task by tense interaction (*p* > 0.05). Tamhane's post hoc statistics indicate that the past, present, and future tenses are all significantly lower in accuracy than neutral tense (*p* < 0.001). Additionally, past tense and present tense were significantly different from each other (*p* = 0.01; mean difference = −0.9; SE = 0.028). The difference between past versus future and present versus future was not statistically significant (*p* > 0.05). Tamhane's post hoc analyses of the effect of experimental tasks showed that the sentence production picture task was significantly less accurate than the three other tasks: sentence production priming, sentence completion, and grammaticality judgment (*p* < 0.001).

In order to more precisely examine tense differences for each task, four separate ANOVAs were computed for each task. An a priori decision was made to use a more conservative *p* value to account for the multiple ANOVAs (one-fourth of 0.05 yielded a significant *p* of <0.0125). No significant differences were found between the tenses for sentence production with pictures (*F*(2,21) = 0.121, *p* = 0.88, *p* > 0.05), grammaticality judgment (*F*(2,59) = 2.8, *p* > 0.05), or sentence completion (*F*(2,134) = 2.7, *p* > 0.05). There was a significant difference across tenses for sentence production priming (*F*(3,159) = 9.3, *p* < 0.001). Post hoc comparisons with Bonferroni correction revealed significantly lower accuracy of past and future sentences compared to neutral sentences (mean difference > 0.25, *p* < 0.0125). Comparisons of present versus neutral (mean difference = 0.2, *p* = 0.04) and past versus present tense (mean difference = 0.17, *p* = 0.02) approached the significance threshold of *p* < 0.0125.

To summarize, the analysis of 143 published datasets revealed an effect of task on overall accuracy: sentence production with pictures yields significantly lower accuracy than the other three experimental tasks. While there was consistent superiority for neutral sentences over tensed sentences, the differences between past, present, and future sentences were inconsistent across tasks and primarily driven by the sentence production priming task.

## 3. Tense Production Using Elicited Picture Description

In order to further inform our understanding of how persons with agrammatic aphasia are affected by verb tense, we examined previously acquired data from participants reported in our prior studies [32, 66–71]. The data reported here are from the intake protocol regularly used to determine the presence of agrammatic aphasia. Although these data were collected previously, these data have not been reported in any prior study or included in the meta-analysis reported in the previous section.

### 3.1. Methods

#### 3.1.1. Participants

Sixteen participants (10 men, 6 women) with a medical history of left cerebrovascular accident were included in the study. The participants ranged in age from 39 to 70 years (mean = 54.06) and, at the time of testing, and ranged from 14 to 163 months from onset of their left hemisphere damage (mean = 52.56). All participants were native speakers of English and premorbidly right-handed (except AP10 who was left-handed). None of the participants reported a history of any significant speech-language, psychiatric, or neurological diagnoses prior to the onset of left hemisphere damage. Demographic and language profiles of all sixteen participants are reported in Table 3. All participants provided informed consent prior to participation in the study.

#### 3.1.2. Background Speech, Language, and Cognitive Information

All participants were given a battery of speech and language tests and screening for hearing, visual, and cognitive status. This included elicitation of narratives of the cookie theft picture and selected narrative story cards [72], Western Aphasia Battery-Revised (WAB-R, [73]), inventory of articulatory characteristics of the Apraxia Battery of Adults [74], verbal and nonverbal agility subtests of the Boston Diagnostic Aphasia Examination-3rd Edition (BDAE-3rd Edition, [75]). All participants had a clinical profile of Broca's aphasia (nonfluent speech, relatively preserved comprehension, and impaired repetition). Four participants (AP8, AP15, AP17, and AP26) were unclassifiable as per the subtest scores of the WAB-R although they had nonfluent speech because they were relatively mildly impaired in terms of overall severity. Narrative speech data revealed a morphosyntactic production profile consistent with agrammatism for all sixteen participants with low proportion of complete sentences, low proportion of grammatically accurate sentences, and high noun : verb ratio (see Table 3). None of the participants had significant verbal apraxia, defined as fewer than four features in the apraxia inventory of Apraxia Battery for Adults-2nd Edition and score higher than 8 on the verbal agility subtest of BDAE-3rd Edition. All participants could successfully read single words and passed a hearing screening at 40 dBHL in both ears (except AP1 who used hearing aids) and a vision screen (at least 20/40 corrected or uncorrected vision in both eyes).

#### 3.1.3. Materials and Procedure

The stimuli consisted of twenty black and white line drawings of transitive (*N* = 15) and intransitive (*N* = 5) action sequences. There were an equal number of regular and irregular verbs (ten each). A sequence of three action pictures, depicting future, present, and past tense, was used (see Figure 2). In order to elicit sentences with different tenses, a variety of temporal adverbs were printed on one of the three pictures in the sequence (*Yesterday*,* In a moment*,* Tomorrow*,* Right now*, and* Nowadays*). The nouns and verb pertaining to the action were also printed on each picture to alleviate the impact of lexical retrieval failure on sentence production. Participants were instructed to describe each picture using a single sentence beginning with the temporal adverb and using the words printed on the picture. Two practice items were provided during which the experimenter clarified the use of present progressive and simple present for “*Right now*” and “*Nowadays*,” respectively. Five sentences each were elicited in the simple present (verb-*s*), present progressive (is verb*-ing*), simple past (verb*-ed*), and future tense (will verb) (total *N* = 20). There was no response time limit and participants were allowed to self-correct spontaneously.

Responses were transcribed by the experimenter during the session and later scored for accuracy. The final self-corrected response was scored as correct if it unambiguously matched the target tense elicited by the appropriate adverb. Hence, a response such as “*Yesterday the girl will peel the potatoes*” is scored as incorrect. Word order errors (e.g., noun-noun-verb as in “*Yesterday…the girl…potatoes…peeling*”) were ignored and only verb morphology was scored for accuracy of tense. In order to be consistent with studies that were included in the meta-analysis of the previous section of this paper, scores of simple present and present progressive were combined to get the present tense score (the pattern of performance across tenses was unchanged irrespective of whether present tense was considered separately (simple present versus present progressive) or when these two scores were combined). Accuracy scores for each participant for each tense were converted to standard (*z*) scores for statistical analysis. Errors were categorized into* incorrect tense* (e.g.,* peels* for* peeled*),* unmarked* in which the verb lacks any clear tense or agreement marking (e.g.,* peeling* or* peel produced without an auxiliary or modal*),* others*, which included omissions of the verb, and* no responses*.

### 3.2. Results

One-way analysis of variance found no main effect of tense (mean (SD) present = 0.22 (0.19), past = 0.19 (0.29), and future = 0.30 (0.43), *F*(2,45) = 0.535, *p* = 0.59). Planned pairwise comparisons yielded no significant differences between any two tense categories (*p* > 0.05). Individual participant data, which are provided in Table 4, show that overall most participants had zero accuracy for at least one tense. Four participants scored 100% on future tense (AP3, AP6, AP10, and AP19), and two participants each scored 100% on present and past tense (AP15 and AP9). The distribution of errors for individual participants is given in Table 5. There was preponderance of* unmarked verb* substitutions (75.4% of all errors) across all participants, with the exception of AP9.* Incorrect tense* errors were produced 15.8% of the time and* other* errors were produced in 8.7% of the instances.

## 4. Discussion

The aim of this study was to further characterize the tense deficit in persons with agrammatic aphasia in terms of differential impairment across temporal reference and experimental task. This was achieved in two ways: by synthesizing the existing published evidence on tense deficits in 143 datasets of persons with agrammatic aphasia and by contributing new data from sixteen participants using a picture description task. The obvious advantage of analyzing a large dataset, as was done in this study, is to minimize the influence of different sources of variability arising in small group studies: individual variability which can skew group averages, language-specific morphosyntactic patterns, and the experimental manipulations (task instructions, stimuli) which could inadvertently impact one experimental condition. There were three main findings of the meta-analysis. First, the aphasic participants were significantly impaired in the experimental tasks, with an overall accuracy of just 47%. Second, we found a significant effect of experimental task on performance accuracy. Third, there was no striking accuracy difference with respect to temporal reference, but with one exception: only in the sentence production priming task was the past tense worse than present and future tenses (by 12 and 14 percent, resp.). The new data from picture description found no difference among tense types, replicating the findings of the meta-analysis. In the following sections, we will consider the question of between-tense differences, followed by a discussion of the current understanding of verb tense impairment in agrammatism.

### 4.1. Is There a Differential Tense Impairment?

**Figure 1:**
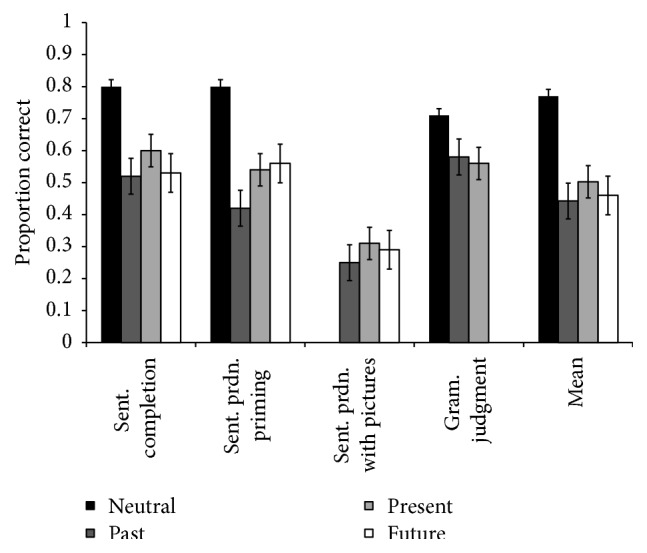
Findings of the meta-analysis showing mean proportion accuracy sorted by tense and experimental task. The number of datasets for each tense is *N* = 42 (neutral), *N* = 143 (past), *N* = 130 (present) and *N* = 81 (future).

**Figure 2:**
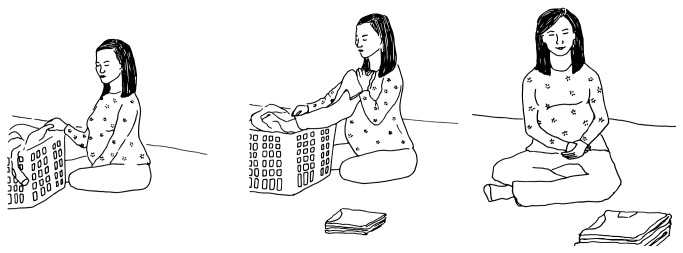
An example of a picture sequence used to elicit different tenses. The picture shows the action “folding.” The action is about to begin in the first (future), ongoing in the second (present), and completed in the third (past) picture of the sequence.

As outlined in the Introduction, there were two compelling reasons to predict worse performance on past and future tense compared to present tense for agrammatic aphasic speakers. The first is a divergence between speaking time and reference time for past and future, but not present tense (Table 1, [44, 45, 47, 57]). Additionally, neurologically healthy speakers process sentences referring to ongoing events faster than completed or hypothetical events [50–55]. And developmentally, children acquire past tense later than present tense [76, 77] and show a past tense disadvantage in specific language impairment [78]. If these differences in temporal reference influenced sentence processing in agrammatism, we should have found a consistent disadvantage for past and future across all experimental tasks. This prediction was not confirmed. Moreover, if one were to argue that differences in processing temporal reference are subtle and observable only when agrammatic persons' syntactic mechanism is sufficiently taxed, then we should have observed the predicted pattern in the most challenging experimental task, sentence production with pictures. Unlike for sentence completion, priming, and grammaticality judgment, producing sentences with pictures task employs syntactic structure building in addition to computation of verb tense [20]. Not surprisingly, performance was 22 to 27 percent worse on sentence production with pictures compared to the other experimental tasks (Figure 1). However, the prediction of* differential* tense performance in this challenging task was not confirmed, both in the meta-analysis and new empirical data. Thus, this study found that although computation of verb tense was generally impaired in agrammatism, there was little variability among tense types for three out of four experimental tasks, including the task that had the lowest overall accuracy.

The reason for the past tense disadvantage specifically in the sentence production priming task is unclear [29, 36, 37, 64]. There are three noteworthy considerations in interpreting this result. First, the magnitude of the past tense disadvantage (12–14%) is relatively modest in comparison to the significant difficulty with computing tense (47% accuracy) in agrammatism. Hence, any theoretical account of agrammatism must account for the overarching tense difficulty foremost before accounting for the relative differences in a specific task. Second, given the broader pattern of a lack of differential tense performance in other experimental tasks, the most parsimonious explanation for the past tense disadvantage is the specific demands of the sentence production priming (SPP) task. The elicitation sentences used in SPP and sentence completion are analogous (e.g., Now the man is… [eating an apple]), but SPP also includes a precedent sentence (e.g., Now the man is peeling an apple) [29]. It is plausible that the need to mentally switch actions (*peeling* to* eating* in this example) increases processing demands, which are then specifically detrimental to completed actions because the stimulus pictures do not actually show the action. For instance, a sentence such as “*The man has poured the milk*” is elicited using a picture of a man sitting in front of a full glass of milk [60, 79]. Two other findings that are consistent with the SPP task-specific interpretation are a disadvantage for perfect aspect using the SPP task in which stimuli did not depict the action [29, 37] and a similar performance disadvantage in fluent (nonagrammatic) aphasia [29]. Further research is necessary to verify whether verb tense production in such pictured elicitation tasks is affected by the consistency with which the action is (not) depicted in the elicitation stimuli.

### 4.2. How Can We Characterize the Verb Tense Impairment in Agrammatism?

Collective data from 122 persons with agrammatic aphasia (PWAA) analyzed in this study show (1) an overwhelming difficulty in production and processing of verb tense, (2) individual differences in accuracy of different tenses (see the appendix and Table 3), and (3) a hierarchy of task difficulty. The first finding reiterates the view that verb tense impairment is a* core* symptom of agrammatism. In fact, not a single PWAA showed unimpaired performance, although a handful scored well above chance (e.g., patients EL and HM in Wenzlaff [15], C7 in Bastiaanse et al. [36]; see the appendix). Other studies have reported that PWAA perform poorly on judgment of verb tense violations (e.g., ^∗^John slept tomorrow), while they are sensitive to judgment of structural syntactic violations (e.g., ^∗^Father sleeping brother) [10, 12, 32, 80]. Further, accuracy of elicited tense production is found to correlate significantly with tense judgment in PWAA [32]. This greater difficulty of tense compared to structural syntactic judgments and correlation with sentence production further supports the centrality of verb tense difficulties to agrammatism.

Numerous theoretical accounts over the past two decades suggest a difficulty in the semantics to morphosyntax interface as the most parsimonious account of verb tense deficits of agrammatism. These include a distinction between interpretable and uninterpretable syntactic features [21, 23, 31], referential and nonreferential syntax [33], encoding message features onto morphosyntax [12], and mapping between functional and grammatical elements [3, 81].

The second noteworthy point of verb tense performance is the intraindividual variability across tense types; that is, many PWAA's accuracy was widely different across tenses (e.g., JS scored 0.3 and 0.8 for past and present tense, [11]; see the Appendix). Without comparisons with a control group, it is unclear whether any of these differences is an actual dissociation [82, 83]. In some instances, the small numbers of stimuli for each tense condition preclude meaningful statistical comparisons to examine neuropsychological dissociations. However, prior investigations have analyzed patterns of intraindividual variability for verb tense. It was found that PWAA's verb tense errors typically favor verb forms that are more accessible. That is, the target verb form is substituted by another verb form that is more frequent (hence more accessible) than the target [42, 84–86]. Further, individual participants tend to overuse a single verb form, giving a near-perfect accuracy for the verb tense corresponding to that verb form ([42]; see also Table 3). For example, AP9 in this study overused the past form (verb + ed) and AP15 overused progressive verbs, resulting in 100% accuracy for these tenses, but close-to-zero accuracy for other tenses (accuracy in Table 3 and substitution errors in Table 4). To summarize, the most likely source of individual variability in tense production is different strategies of verb form accessibility adopted by individual participants. That is, when faced with the challenge of transitioning from semantic representations to morphosyntactic representations, the agrammatic system resorts to using the most accessible elements. While the current data cannot directly address tense dissociations, future research comparing performance with a normal control group could help elucidate whether there are any dissociations among tense types.

Finally, we found a robust effect of task difficulty on accuracy, with significantly worse performance on overt production (sentence production priming and sentence production with pictures) compared to selection (grammaticality judgment and sentence completion). This task differential is consistent with the computational accounts of agrammatism proposed by Avrutin [38] and Kok et al. [20]. These accounts propose that mapping of conceptual-semantic representations onto morphosyntax involved in tense marking is challenging for PWAA, primarily because it involves more computations and strictly structural operations such as verb agreement. Further, the number of linguistic computations has an additive effect on performance [20]. It is noteworthy that Kok et al. [20] observed that a hierarchy of task difficulty is seen for most, but not all, PWAA (e.g., patients NU and WO in their study). Hence, computational load is one factor (but not the entire source) of the symptom complex of agrammatism.

### 4.3. Conclusions

The data accrued from this study and the past three decades of agrammatism research emphasize that difficulties in processing and producing verb tense are best considered as one of the crucial components of the symptom profile of agrammatism. While mapping of conceptual-semantic representations onto morphosyntax for tense marking is particularly challenging, this is likely to be mediated by processing limitations and accessibility of specific verb forms. At present, there is little evidence of a differential tense impairment; however, future research specifically aimed at examining double dissociations in individual participants and the effect of stimulus manipulations may shed more light on tense differentials. It is also crucial to establish whether a disadvantage for temporally complete events is a general pattern found across several populations or is a core characteristic of agrammatic aphasia, because a parsimonious theory of agrammatism really needs to account for symptoms that are unique to the condition. Data from other populations indicate that the disadvantage for past events is not unique to agrammatism and is found in healthy adults and children, fluent aphasia, and specific language impairment [29, 50–55, 76–78].

## Figures and Tables

**Table 1:** Temporal distance (—) between speaking time (S), reference time (R), and event time (E).

Tense/aspect	Example	Linear ordering
Simple past	He signs the papers	E, R—S
Simple present	He signed the papers	S, E, R
Simple future	He will sign the papers	S—R, E
Present progressive	He will be signing the papers	S, E~, R
Past progressive	He was signing the papers	E~, R—S
Present perfect	He has signed the papers	E—S, R
Past perfect	He had signed the papers	E—R—S

**Table 2:** Individual participant data from the studies included in the meta-analysis.

Reference	Language	Task and response	Patient	Age/gender	MPO	Lesion/aphasia	Number correct/total			
							Neutral	Past	Present	Future
Bastiaanse et al. (2004), page 129 (Table 1) [64]	Dutch	SC, verbal	B1	NR	NR	NR/Broca's		14/30	18/30	
			B2	NR	NR	NR/Broca's		15/30	21/30	
			B3	NR	NR	NR/Broca's		18/30	10/30	

Bastiaanse et al. (2011), pages 671-672 (Appendices 3 and 4) [36]	Chinese	SPP, verbal	C1	42/M	127	LCVA/Broca's	20/20	11/20	1/20	1/20
			C2	22/M	96	TBI/Broca's	20/20	12/20	6/20	6/20
			C3	50/M	97	LCVA/Broca's	19/20	13/20	13/20	18/20
			C4	41/M	180	LCVA/Broca's	18/20	0/20	1/20	0/20
			C5	55/M	92	LCVA/Broca's	20/20	10/20	7/20	16/20
			C6	65/M	204	LCVA/Broca's	20/20	2/20	0/20	0/20
			C7	33/M	125	LCVA/Broca's	16/20	16/20	20/20	20/20
			C8	55/M	156	LCVA/Broca's	17/20	5/20	8/20	8/20
			C10	50/M	177	LCVA/Broca's	20/20	0/20	0/20	0/20
			C11	51/M	212	LCVA/Broca's	11/20	5/20	6/20	4/20
	English	SPP, verbal	E1	52/M	59	LCVA/Broca's	16/20	13/20	19/20	19/20
			E2	47/M	55	LCVA/Broca's	17/20	18/20	16/20	20/20
			E3	64/M	220	LCVA/Broca's	19/20	3/20	13/20	12/20
			E4	48/F	23	LCVA/Broca's	3/20	12/20	19/20	12/20
			E5	53/M	108	LCVA/Broca's	20/20	12/20	18/20	19/20
			E6	60/F	61	LCVA/Broca's	19/20	6/20	4/20	7/20
			E7	53/M	43	RCVA/Broca's	20/20	14/20	19/20	19/20
			E8	68/M	180	TBI/Broca's	3/20	4/20	12/20	4/20
			E9	74/F	36	LCVA/Broca's	16/20	6/20	20/20	19/20
			E10	54/M	39	LCVA/Broca's	18/20	17/20	16/20	13/20
			E11	58/M	226	LCVA/Broca's	16/20	12/20	20/20	20/20
			E12	37/M	34	LCVA/Broca's	4/20	2/20	12/20	3/20
	Turkish	SPP, verbal	T1	68/M	2	LCVA/Broca's		6/20	17/20	17/20
			T2	54/M	5	LCVA/Broca's		4/20	9/20	7/20
			T3	49/F	84	LCVA/Broca's		11/20	15/20	18/20
			T4	43/F	4	LCVA/Broca's		10/20	20/20	15/20
			T5	68/M	1	LCVA/Broca's		11/20	16/20	18/20
			T6	39/F	7	LCVA/Broca's		10/20	16/20	18/20
			T7	65/M	12	LCVA/Broca's		6/20	4/20	17/20
			T8	59/M	2	LCVA/Broca's		13/20	18/20	18/20

Clahsen and Ali (2009), page 446 (Table 6) [11]	English	SC, pointing	BG	36/M	60^*∗*^	NR/Broca's		8/10	10/10	
			JS	65/M	96^*∗*^	NR/Broca's		2/10	8/10	
			KC	78/M	96^*∗*^	NR/Broca's		3/10	6/10	
			RC	77/M	24^*∗*^	NR/Broca's		8/10	6/10	
			JP	68/M	60^*∗*^	NR/Broca's		10/10	7/10	
			KS	66/M	18^*∗*^	NR/Broca's		9/10	6/10	
			PB	82/M	36^*∗*^	NR/Broca's		6/10	5/10	
			BM	52/M	36^*∗*^	NR/Broca's		9/10	5/10	
			BR	82/M	36^*∗*^	NR/Broca's		6/10	3/10	
		GJ, verbal	BG			NR/Broca's		15/20	15/20	
			JS			NR/Broca's		11/20	12/20	
			KC			NR/Broca's		13/20	12/20	
			RC			NR/Broca's		12/20	11/20	
			JP			NR/Broca's		18/20	16/20	
			KS			NR/Broca's		13/20	14/20	
			PB			NR/Broca's		17/20	11/20	
			BM			NR/Broca's		11/20	11/20	
			BR			NR/Broca's		15/20	14/20	

Dickey et al. (2008) [61]	English	GJ, keyboard	A01	60/F	180^*∗*^	NR/Broca's	27/30	26/60	16/30	11/30
			A02	63/F	132^*∗*^	NR/Broca's	28/30	44/60	29/30	29/30
			A03	56/F	168^*∗*^	NR/Broca's	26/30	36/60	14/30	15/30
			A04	57/F	48^*∗*^	NR/Broca's	21/30	27/60	14/30	15/30
			A05	50/M	108^*∗*^	NR/Broca's	18/30	24/60	14/30	14/30
			A06	36/F	36^*∗*^	NR/Broca's	19/30	30/60	14/30	14/30
			A07	68/F	144^*∗*^	NR/Broca's	23/30	32/60	14/30	16/30
			A08	66/F	84^*∗*^	NR/Broca's	23/30	39/60	16/30	18/30
			A09	57/M	48^*∗*^	NR/Broca's	7/30	4/60	4/30	4/30
			A10	36/F	24^*∗*^	NR/Broca's	24/30	37/60	14/30	14/30

Dragoy and Bastiaanse (2013), page 127 (Appendix 3) [29]	Russian	SPP, verbal	1	31/F	35	NR/nonfluent		27/40	15/20	8/20
			2	32/F	29	NR/nonfluent		31/40	11/20	10/20
			3	33/F	20	NR/nonfluent		20/40	12/20	5/20
			4	35/M	70	NR/nonfluent		23/40	17/20	5/20
			5	36/F	29	NR/nonfluent		19/40	17/20	10/20
			6	46/M	16	NR/nonfluent		20/40	14/20	15/20
			7	68/M	29	NR/nonfluent		20/40	15/20	18/20

Duman and Bastiaanse (2008), page 9 (Appendix A)[37]	Turkish	SC, verbal	B1	66/F	2.5	LCVA/agrammatic		16/30		15/30
			B2	70/M	6	LCVA/agrammatic		4/30		18/30
			B3	44/F	16	LCVA/agrammatic		14/30		13/30
			B4	47/F	26	LCVA/agrammatic		11/30		12/30
			B5	40/M	28	LCVA/agrammatic		13/30		15/30
			B6	26/F	120	TBI/agrammatic		16/30		16/30
			B7	75/M	20	LCVA/agrammatic		3/30		19/30

Faroqi-Shah and Thompson (2004), page 492 (Figure 3) [42]	English	SPPi, verbal	CH	56/M	90	LCVA/Broca's		2/17	14/17	1/17
			MK	54/M	12	LCVA/Broca's		3/17	0/17	7/17
			MR	44/F	45	LCVA/Broca's		3/17	13/17	2/17
			JP	65/M	30	LCVA/Broca's		1/17	2/17	10/17
			MD	62/M	120	LCVA/Broca's		3/17	9/17	3/17
			JO	69/M	88	LCVA/Broca's		7/17	3/17	7/17
			RH	64/M	100	LCVA/Broca's		9/17	2/17	0/17
			LD	52/F	14	LCVA/Broca's		6/17	0/17	10/17

Faroqi-Shah and Thompson (2007), pages 139-140 (Figures 2 and 3) [12]	English	SC, verbal	B1	55/M	156^*∗*^	LCVA/Broca's	11/15	19/30	14/15	
			B2	58/M	48^*∗*^	LCVA/Broca's	15/15	18/30	14/15	
			B3	59/M	168^*∗*^	LCVA/Broca's	13/15	17/30	5/15	
			B4	64/M	60^*∗*^	LCVA/Broca's	10/15	17/30	8/15	
			B5	55/F	108^*∗*^	LCVA/Broca's	11/15	14/30	5/15	
			B6	68/M	120^*∗*^	LCVA/Broca's	9/15	11/30	5/15	
			B7	59/F	96^*∗*^	LCVA/Broca's	12/15	12/30	10/15	
			B8	63/M	108^*∗*^	LCVA/Broca's	12/15	20/30	5/15	
			B9	66/M	60^*∗*^	LCVA/Broca's	14/15	14/30	8/15	
			B10	55/F	72^*∗*^	LCVA/Broca's	15/15	11/30	5/15	
		SC, verbal	B1			LCVA/Broca's		3/15	10/15	7/15
			B2			LCVA/Broca's		5/15	8/15	8/15
			B3			LCVA/Broca's		10/15	8/15	9/15
			B4			LCVA/Broca's		5/15	9/15	10/15
			B5			LCVA/Broca's		7/15	10/15	7/15
			B6			LCVA/Broca's		8/15	7/15	7/15
			B7			LCVA/Broca's		8/15	5/15	8/15
			B8			LCVA/Broca's		8/15	8/15	9/15
			B9			LCVA/Broca's		3/15	9/15	8/15

Fyndanis et al. (2012), page 1140 (Table 4) [31]	Greek	SC, verbal	GT	44/M	4.5	LCVA/agrammatic		8/21	12/16	12/19
			GL	59/M	38	LCVA/agrammatic		2/21	8/16	9/19

Jonkers and de Bruin (2009), page 1265 (Appendix 2) [65]	Dutch	SC, verbal	B1	80/M	26	NR/Broca's		16/20	14/20	
			B2	70/M	12	NR/Broca's		2/20	16/20	
			B3	41/F	4	NR/Broca's		10/20	19/20	
			B4	55/M	3	NR/Broca's		19/20	19/20	
			B5	41/M	49	NR/Broca's		5/20	2/20	
			B6	78/M	42	NR/Broca's		11/20	20/20	
			B7	41/F	4	NR/Broca's		16/20	13/20	

Nanousi et al. (2006), pages 220, 223, and 226 (Tables 6, 8, and 10) [21]	Greek	SPP, verbal	DS	66/M	48^*∗*^	LCVA/Broca's		31/60	25/60	15/30
			PA	61/M	48^*∗*^	LCVA/Broca's		29/60	32/60	13/30
			ZA	41/M	36^*∗*^	LCVA/Broca's		25/60	19/60	9/30
			AS	38/M	96^*∗*^	LCVA/Broca's		28/60	28/60	15/30
			AJ	55/M	72^*∗*^	LCVA/Broca's		23/60	19/60	10/30
			RS	46/M	108^*∗*^	LCVA/Broca's		25/60	25/60	12/30
		SPP, verbal	DS			LCVA/Broca's		19/60		21/60
			PA			LCVA/Broca's		23/60		32/60
			ZA			LCVA/Broca's		11/60		20/60
			AS			LCVA/Broca's		23/60		33/60
			AJ			LCVA/Broca's		2/60		18/60
			RS			LCVA/Broca's		15/60		25/60
		SC, verbal	DS			LCVA/Broca's		26/48	34/48	13/24
			PA			LCVA/Broca's		34/48	35/48	11/24
			ZA			LCVA/Broca's		25/48	27/48	12/24
			AS			LCVA/Broca's		30/48	32/48	16/24
			AJ			LCVA/Broca's		26/48	30/48	10/24
			RS			LCVA/Broca's		29/48	27/48	14/24

Wenzlaff and Clahsen (2004), page 64 (Table 4) [15]	German	SC, verbal	DB	58/F	276^*∗*^	LCVA/Broca's		14/20	14/20	
			EL	49/F	264^*∗*^	LCVA/Broca's		19/20	14/20	
			KM	84/F	24^*∗*^	LCVA/Broca's		12/20	17/20	
			MH	59/M	180^*∗*^	LCVA/Broca's		12/20	15/20	
			HM	66/M	144^*∗*^	LCVA/Broca's		17/20	13/20	
			WH	70/M	192^*∗*^	LCVA/Broca's		8/20	17/20	
			OP	69/M	24^*∗*^	LCVA/Broca's		12/20	7/20	
		GJ, verbal	DB			LCVA/Broca's		10/20	10/20	
			EL			LCVA/Broca's		10/20	10/20	
			KM			LCVA/Broca's		12/20	11/20	
			MH			LCVA/Broca's		12/20	11/20	
			HM			LCVA/Broca's		18/20	17/20	
			WH			LCVA/Broca's		10/20	10/20	
			OP			LCVA/Broca's		10/20	10/20	

GJ: grammaticality judgment; LCVA: left cerebrovascular accident; MPO: months post onset; NR: not reported; RCVA: right cerebrovascular accident; SC: sentence completion; SPP: sentence production priming; SPPi: sentence production with picture description; TBI: traumatic brain injury; ^*∗*^months post onset calculated from years in the original study.

**Table 3:** Demographic and language data of participants in the sentence production task.

Patient	Age, gender, hand	Educ. years	MPO	Western Aphasia Battery-Revised					Narrative speech analysis					
				Aphasia	AQ	Spont. Speech	Compr.	Naming	WPM	MLU	Prop. Sent.	Prop. Gram. Sent.	Open : Closed	Noun : Verb
AP1	59/M/R	24	29	Broca's	66.8	13	7.6	6.4	39.04	4.08	0.43	0.11	1.09	0.96
AP3	65/M/R	17	73	Broca's	65.9	11	8.8	6.1	24.67	5.76	0.52	0.31	1.15	1.7
AP5	64/F/R	19	75	Broca's	57.2	11	7.6	5.3	48.24	2.78	0.52	0.35	2.29	2.75
AP6	70/F/R	17	163	Broca's	65.9	14	6.35	7.8	53.44	3.68	0.22	0.1	1.26	5.25
AP8	55/F/R	17	114	Mixed	90.4	18	10	8.6	34.41	5.88	0.84	0.57	1.07	2.06
AP9	56/M/R	14	23	Broca's	83.6	15	9.5	7	60.3	5.89	0.8	0.63	0.9	1.87
AP10	40/M/L	18	115	Broca's	77.4	14	8.5	8.4	29.19	3.83	0.48	0.5	1.35	1.93
AP12	61/M/R	16	15	Broca's	57.2	11	6.6	6.5	68	5.4	0.76	0.36	0.67	0.61
AP14	44/M/R	19	14	Broca's	71.6	13	9.3	7.6	32.56	1.95	0.2	0	5	1.41
AP15	55/M/R	13	16	Mixed	80.5	13	9.45	8.6	75.82	3.41	0.36	0.39	1.8	4.23
AP17	44/F/R	17	15	Mixed	45.4	7	6.3	6	11.11	1.67	0.1	0	58	6.57
AP18	39/M/R	18	26	Broca's	71.8	13	6.8	8.5	36	3.16	0.36	0.5	1.1	5
AP19	55/M/R	19	14	Broca's	61.8	11	9.2	6.5	23.12	1.64	0.1	0	4.07	3.67
AP23	47/M/R	12	33	Broca's	68.8	11	7.9	7.1	13.76	2.68	0.18	0.44	1.18	1.79
AP24	62/F/R	15	61	Broca's	61.2	12	6.25	5.6	28.09	2.93	0.5	0.16	2.3	2.47
AP26	49/F/R	14	55	Mixed	80.3	15	9.05	8.5	31.63	31.24	0.1	0	6.1	5.5

AQ: aphasia quotient (max = 100); Compr.: comprehension score (max = 10), Noun : Verb: ratio of nouns to verbs; MLU: mean length of utterance in words; MPO: months post onset, Open : Closed: ratio of open to closed class words; Prop. Gram. Sent.: proportion of grammatically accurate sentences; Prop. Sent.: proportion of sentences; Spont. Speech: spontaneous speech; WPM: words per minute.

**Table 4:** Individual tense accuracy data for sentence production task.

Participant	Past	Present		Future
		Simple	Progressive	
AP1	0.4	0.8	0.0	0.0
AP3	0.0	0.0	0.2	1.0
AP5	0.0	0.0	0.0	0.0
AP6	0.0	0.0	0.0	1.0
AP8	0.6	0.0	0.6	0.2
AP9	1.0	0.0	0.2	0.0
AP10	0.0	0.3	0.0	1.0
AP12	0.2	0.0	0.4	0.0
AP14	0.0	0.0	0.8	0.4
AP15	0.0	0.0	1.0	0.0
AP17	0.0	0.0	0.6	0.2
AP18	0.2	0.0	0.0	0.0
AP19	0.0	0.0	0.2	1.0
AP23	0.4	0.4	0.2	0.0
AP24	0.2	0.0	0.0	0.0
AP26	0.0	0.6	0.6	0.0

Mean (SD)	0.19 (0.28)	0.22 (0.3)		0.30 (0.4)

**Table 5:** Distribution of errors for each participant in the sentence production task.

Participant	Incorrect tense	Unmarked	Others	Total errors
AP1	7	7	0	14
AP3	0	13	1	14
AP5	0	19	1	20
AP6	0	14	1	15
AP8	4	8	1	13
AP9	12	1	1	14
AP10	4	3	0	7
AP12	5	9	3	17
AP14	0	10	4	14
AP15	0	15	0	15
AP17	0	15	1	16
AP18	2	16	1	19
AP19	0	11	3	14
AP23	3	9	3	15
AP24	1	17	1	19
AP26	0	14	0	14
